# Efficacy and compliance of an administrative MDT-driven antimicrobial prophylaxis protocol in Class I incisions: a retrospective-prospective study

**DOI:** 10.3389/fpubh.2026.1794933

**Published:** 2026-04-22

**Authors:** Yusen Li, Jixia Wang, Luhan Wen, Ning Li, Juan Wang, Zhen Liu, Xing Zhang, Kaiyang Fan, Xiujuan Meng

**Affiliations:** 1 Healthcare-Associated Infection Control Department, Affiliated Hospital of Jining Medical University, Jining, Shandong, China; 2 Department of Pharmacy, Affiliated Hospital of Jining Medical University, Jining, China; 3 Medical Department, Affiliated Hospital of Jining Medical University, Jining, China

**Keywords:** administrative MDT, antibacterial drug, Class I incision, perioperative period, prevention

## Abstract

**Background:**

Perioperative antibiotic prophylaxis plays a key role in preventing surgical site infections (SSIs). However, inappropriate use may increase the risk of antimicrobial resistance and SSIs. This study aims to evaluate the use of perioperative prophylactic antibiotics in Class I incisions after implementing administrative multidisciplinary team (MDT) interventions and assess the effectiveness of these interventions in improving management practices.

**Methods:**

Retrospective collection of perioperative data from 2017 served as baseline data. The administrative intervention was divided into three phases: Phase I: The administrative Multi-departmental team (MDT) was composed of Medical Affairs Department, Hospital Infection Management Department, and Pharmacy Department (January 2018–December 2019). During this phase, the three departments will collaboratively conduct surveillance, evaluation, and public reporting on perioperative antimicrobial prophylaxis for Class I (clean) incisions and organize targeted training initiatives based on the findings. Phase II: The “administrative MDT” model will now be comprehensively expanded and integrated into key areas of our healthcare system, encompassing the Nursing department, Operating room, Anesthesiology department, and various clinical units. This strategic development will span a multi-department joint intervention phase, commencing from January 2020 and concluding in December 2022; Simultaneously, targeted improvement measures were implemented to address issues identified in Phase I. Phase III: Continuous follow-up (January 2023–December 2024), To evaluate the improvement effectiveness of each intervention phase compared to the baseline monitoring results.

**Results:**

After administrative interventions, all monitoring indicators for prophylactic antibiotic use in Class I incisions showed significant improvement: The prophylactic antibiotic use rate for Class I incisions decreased from 33.72% (7,168/21259) at baseline to 29.01% (24,314/83804) in Phase II and further to 25.91% (19,757/76259) in Phase III, which were statistically significant different (*p* = 0.000 < 0.05). The average score rate of surgical staff on antibiotic-related knowledge assessments increased from 70.83% at baseline to 96.33% in Phase II, and high at 95.41% in Phase III. The incidence of Class I incision infections decreased slightly from 0.11% at baseline to 0.09% in Phase II, and to 0.10% in Phase III (No statistically significant difference). The results demonstrate that MDT intervention was associated with a decrease in antimicrobial use, and no significant increase in surgical site infection (SSI) rates was observed.

**Conclusion:**

This study applied the “Administrative MDT Model” to the management of prophylactic antibiotic use in Class I incisions, As a result, the utilization rate of prophylactic antibiotics for these procedures was significantly reduced. The “Administrative MDT Model,” effectively reduced unnecessary antibiotic use while maintaining low infection rates.

## Background

Perioperative period generally refers to the time from the start of treatment related to the surgery to the end of treatment related to the surgery, including three stages: preoperative, intraoperative, and postoperative. In 2015, the National Health and Family Planning Commission revised the “Guidelines for Clinical Application of Antibacterial Drugs,” which made clear regulations on the prophylactic use of antibacterial drugs during the perioperative period for surgical patients. For patients undergoing Class I incision surgeries, antibacterial drugs are not generally required for prophylaxis against infection during the perioperative period. However, there is a relative lack of knowledge about the clinical use of antibacterial drugs during the perioperative period, along with a lack of strong regulation, leading to a high rate of antibacterial drug use. At the same time, the phenomenon of irrational use of antibacterial drugs is still widespread, which directly affects the level of rational drug use and the quality of medical care in hospitals (1–3).

The MDT (Multi-Disciplinary Treatment) model was first proposed by the Mayo Clinic in the United States in the 1960s. It is a process where multidisciplinary senior experts discuss together to develop personalized treatment plans for patients. In recent years, researchers have applied the concept of multidisciplinary teams (MDT) to fields such as disease diagnosis and treatment, achieving good results (4–6). The management of prophylactic use of antibacterial drugs during the perioperative period for Class I incision surgeries involves multiple departments, including the medical department, nursing department, pharmacy department, and hospital infection control department. Therefore, this study applied the “administrative MDT” concept to the management of prophylactic use of antibacterial drugs during the perioperative period for Class I incision surgeries and achieved significant results.

## Materials and methods

### Study design

Baseline data (2017): In 2017, perioperative antimicrobial use for Class I incision surgeries was managed by the Hospital Infection Management Department. Due to the lack of a systematic management mechanism across multiple departments, the perioperative medication data of Class I incision surgeries in 2017 were used as baseline data. The collected data included:

- Surgical start time and end time of Class I incision surgery patients;
- Class of antimicrobial agents used;
- Infusion start and end times of antimicrobial agents;
- Assessment scores of surgeons on antimicrobial knowledge;
- Incidence of Class I incision infections;

Phase I (January 2018–December 2019): Establishment of the Class I Incision Perioperative Antimicrobial Special Working Group (Version 1.0):

- Leadership: Led by the Vice President in charge of hospital infection management;
- Secretariat: Hosted by the Hospital Infection Management Department;
- Membership: Comprised of heads of the Medical Department, Hospital Infection Management Department, and Pharmacy Department, along with relevant business backbones from these departments.

**Figure 1:**
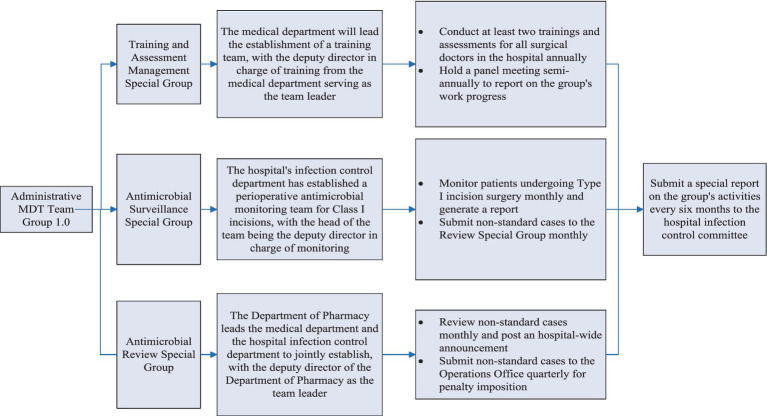
Composition and responsibilities of the administrative MDT team version 1.0.

**Figure 2:**
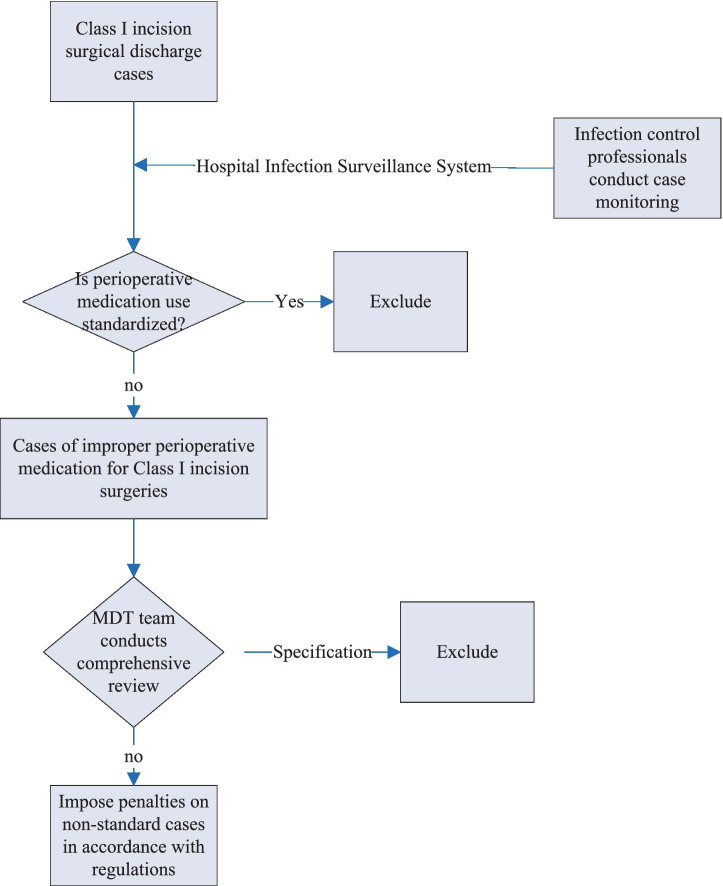
Workflow of the specialized working group on perioperative antibiotics for Class I incisions in stage 1.

The working group consisted of three specialized subgroups and the process (Figures 1, 2):

- Training and Assessment Special Management Subgroup;
- Antimicrobial Surveillance Special Group;
- Antimicrobial Review Special Group.

The responsibilities of the specialized subgroups are shown in Figure 1. Additionally, the Operational Procedures for Perioperative Prophylactic Use of Antimicrobial Agents was revised to clarify the principles and evaluation criteria for prophylactic use of antimicrobial agents in Class I incision surgeries (Table 1).

**Table 1:** Criteria for evaluating the rationality of antibiotic use prevention.

Entry	Reasonable	Unreasonable
Timing of medication	1. Administer medication 0.5 to 1 h before skin incision or at the start of anesthesia, and begin surgery after the infusion is completed; 2. Vancomycin or fluoroquinolones should be administered 1 to 2 h before surgery;	Administration of medication not within specified requirements
Types of medicines	Select antibiotic agents in accordance with the requirements of the hospital’s “Operating Procedures for the Perioperative Prevention and Application of Antibiotics”;	Improper selection of antimicrobial drug varieties
Course of medication	Principles do not exceed 24 h, heart surgery can be extended to 48 h	Over 24 h, with no special instructions given

In the event of special cases, the review of perioperative antimicrobial drugs by Class I incision should be conducted by a centralized review group and the reasons should be noted.

Phase II (January 2020–December 2022): Upgrade to Version 2.0 of the Special Working Group: On the basis of Phase I, the Perioperative Rational Drug Use Surveillance Special Group was added to the working group. Members included:

- Heads of the Nursing Department;
- Head nurses of surgical wards involving Class I incisions (including Spine Surgery, Trauma Orthopedics, Hand and Foot Surgery, Joint Surgery, Operating Room, etc.).

The Hospital Infection Management Department’s special responsible person provided targeted training to the Operating Room and surgical wards to ensure homogeneity in monitoring activities.

**Figure 3:**
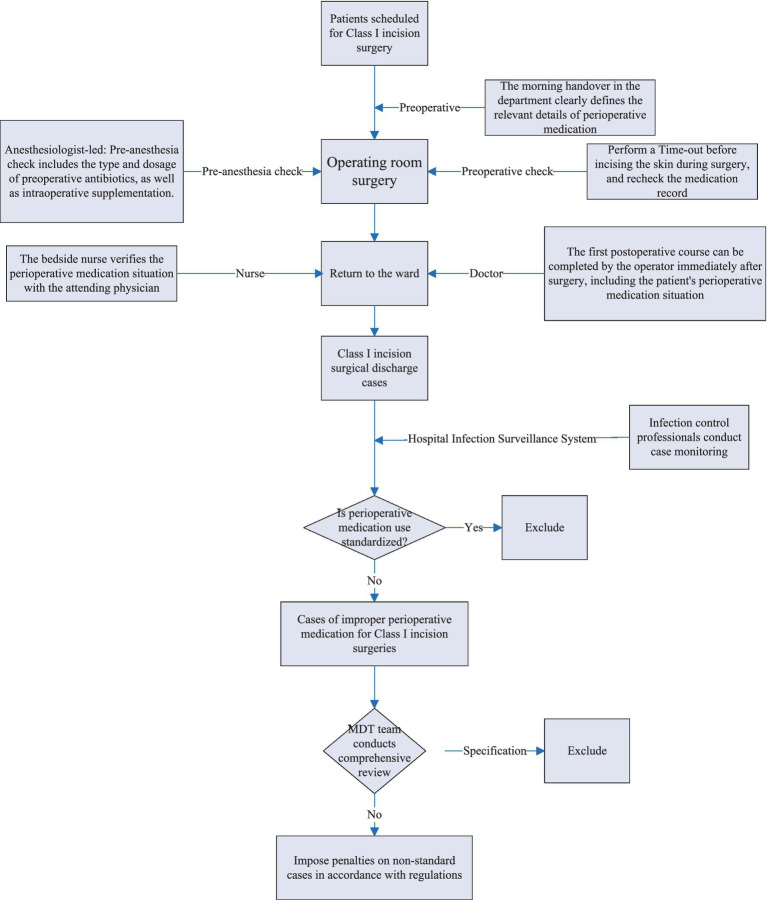
Workflow of the specialized working group on perioperative antibiotics for second phase Class I incisions.

Responsibilities of the Perioperative Rational Drug Use Surveillance Special Group and the process (Figure 3):

- Ward head nurses and bedside nurses (pre- and post-operation) should remind doctors of matters related to perioperative drug selection, duration, etc., which must be documented in the nursing record sheet;
- Operating room head nurses and circulating nurses should remind doctors of perioperative medication requirements. If requirements are not met, surgery can be halted; if surgery is necessary, specific reasons must be recorded in the surgical verification form and signed by the surgeon.

Phase III (January 2023–December 2024): continuous tracking stage: Building on Phase II, the working group continued to track and evaluate perioperative antimicrobial use.

### Statistical analyses

Data were analyzed using SPSS 20.0 statistical software. Quantitative data were presented as mean (score rate); categorical data were expressed as percentages (%), and chi-square tests were used for analysis. A two-tailed *p*-value of less than 0.05 was considered statistically significant.

## Results

### Basic characteristics of patients at different stages

Comparison of age composition (age ≥ 75 years vs. age < 75 years), presence of diabetes, and operative time (> 3 h vs. ≤ 3 h) among patients at various stages revealed no statistically significant differences, as presented in Table 2.

**Table 2:** Comparison of basic characteristics among patients at different stages.

Variable	Baseline data (21259)	Phase I (48608)	Phase II (83804)	Phase III (76259)	Χ^2^	*p*
Age [*n* (%)]					2.604	0.457
Age ≥ 75 years	1,047 (4.92)	2,341 (4.82)	4,093 (4.88)	3,819 (5.01)		
Age < 75 years	20,212 (95.08)	46,267 (95.18)	79,711 (95.12)	72,440 (94.99)		
Diabetes mellitus [*n* (%)]					5.258	0.154
Yes	2,312 (10.88)	5,103 (10.50)	9,012 (10.75)	8,310 (10.90)		
No	18,947 (89.12)	43,505 (89.50)	74,792 (89.25)	67,949 (89.10)		
Operative time [*n* (%)]					6.525	0.089
>3 h	1,061 (4.99)	2,376 (4.89)	3,947 (4.71)	3,497 (4.59)		
≤3 h	20,998 (95.01)	46,232 (95.11)	79,857 (95.29)	72,762 (95.41)		

### Assessment of knowledge on antimicrobial agents in clinical surgery departments

Due to differences in examination methods across phases, the final comparison was based on the score rate of perioperative-related knowledge. The scoring rate at each stage shows an upward trend, as detailed in Table 3.

**Table 3:** Evaluation of antibiotic-related knowledge among surgeons in surgical departments.

Time period	Number of examinees	Total score	Average score	Scoring rate
Baseline data	416	30	21.25	70.83
Phase I	939	40	34.23	85.58
Phase II	1994	100	96.33	96.33
Phase III	1,506	100	95.41	95.41

### Use of antibiotics for prophylaxis in Class I incision surgeries

After the implementation of the administrative MDT working team for perioperative standardized medication use in Class I incisions, the antimicrobial prophylaxis rate for Class I incision surgeries in the whole hospital decreased from 33.72% in Baseline to 30.94% in the first phase, 29.01% in the second phase, and 25.91% in the third phase, with statistically significant differences observed (*p* < 0.05), as detailed in Table 4.

**Table 4:** Post-implementation of administrative MDT work, situation of prophylactic use of antibiotics for Class I incision surgeries.

Time period	Class I incision surgery cases	Prophylactic antibiotics	Prophylactic use rate of antimicrobial agents for Class I incision surgeries (%)	Χ^2^	*p*
Baseline data	21,259	7,168	33.72		
Phase I	48,608	15,038	30.94	52.73	0.000
Phase II	83,804	24,314	29.01	178.84	0.000
Phase III	76,259	19,757	25.91	507.31	0.000

Chi-square tests were performed to compare the first, second, and third phases with the baseline data.

### The usage and utilization rate of prophylactic antibiotics in the top ten departments with the highest number of Class I incision surgeries

After the implementation of the administrative Multi-disciplinary Team (MDT) intervention, the prophylactic antimicrobial use rates in Cardiac Surgery, Neurosurgery, Trauma Orthopedics, Ophthalmology, Urology, and Gynecology showed a significant decrease, with statistically significant differences observed. In contrast, the prophylactic antimicrobial use rates in Bone and Joint Surgery, Spine Surgery, and Breast and Thyroid Surgery initially increased and then decreased, with statistically significant differences noted. For details, see Table 5.

**Table 5:** The improvement status of the top ten departments in terms of the number and usage rate of antimicrobial prophylaxis for Class I incision surgeries.

Department	Baseline data		First Phase		Χ^2^	*p*	Second Phase		Χ^2^	*p*	Third phases		Χ^2^	*p*
	Class I incision surgery cases	Utilization rate	Class I incision surgery cases	Utilization rate			Class I incision surgery cases	Utilization rate			Class I incision surgery cases	Utilization rate		
Cardiac Surgery	1,547	81.97	2,424	68.15	92.58	0	3,196	66.27	125.72	0	2048	70.95	58.14	0
Orthopedic Surgery	1971	76.41	5,206	79.35	7.35	0.01	11,168	78.04	2.57	0.11	12,451	74.44	3.47	0.062
Neuro Surgery	1,076	65.61	2,536	56.82	24.22	0	3,659	39.55	227.87	0	1,668	36.15	227.58	0
Trauma Surgery	1,202	57.65	2,761	47.19	36.66	0	5,140	35.1	207.65	0	4,769	24.85	479.23	0
Spinal Surgery	2074	50.53	5,367	50.25	0.05	0.83	9,876	52.99	4.15	0.04	9,048	48.10	3.99	0.046
Department of Hand and Foot Surgery	1,133	48.19	2,355	48.37	0.01	0.92	4,232	44.85	4.02	0.05	3,877	41.01	18.48	0
Gynecology	1871	14.27	3,335	12.62	2.83	0.09	4,306	12.94	2.01	0.16	3,156	6.59	80.97	0
Ophthalmology	3,185	14.19	7,400	17.35	16.22	0	11,081	8.09	107.4	0	10,989	0.72	1242.97	0
Department of Urology	738	6.23	1,543	2.07	26.15	0	2028	0.54	86.82	0	1,462	0.68	60.88	0
Breast and Thyroid Surgery	2,117	5.67	5,696	7.95	11.85	0	12,185	5.95	0.26	0.61	13,838	2.72	52.73	0

Chi-square tests were performed to compare the first, second, and third phases with the 2017 baseline data.

As shown in Table 4, after the implementation of the administrative Multi-disciplinary Team (MDT) intervention for perioperative prophylactic antimicrobial use in Class I incision surgeries, the prophylactic antimicrobial use rates in Cardiac Surgery, Neurosurgery, Trauma Orthopedics, Ophthalmology, Urology, and General Surgery showed a significant decrease, with statistically significant differences observed (all *p* < 0.05), indicating a marked effect of the administrative MDT intervention. The prophylactic antimicrobial use rate in Gynecology showed a declining trend, but the difference was not statistically significant (χ^2^ = 3.02, *p* = 0.16 > 0.05). In contrast, the prophylactic antimicrobial use rates in Bone and Joint Surgery, Spine Surgery, and Breast and Thyroid Surgery exhibited an upward trend, with statistically significant differences:

- For Bone and Joint Surgery and Breast and Thyroid Surgery, the first phase showed a significant increase (Bone and Joint Surgery: χ^2^ = 7.35, *p* = 0.01 < 0.05; Breast and Thyroid Surgery: χ^2^ = 11.85, *p* = 0.00 < 0.05). Additionally, Breast and Thyroid Surgery showed a significant decrease in the third phase (χ^2^ = 52.73, *p* = 0.00 < 0.05).
- For Spine Surgery, there was no significant change in the first phase compared to the baseline, but a significant increase was observed in the second phase (χ^2^ = 4.15, *p* = 0.04 < 0.05). A significant decrease was also noted in the third phase (χ^2^ = 3.99, *p* = 0.046 < 0.05).

### Class I incision surgeries—rational use of antimicrobial prophylaxis

As shown in Table 5, after the implementation of the administrative Multi-disciplinary Team (MDT) intervention for perioperative prophylactic antimicrobial use in Class I incision surgeries:

- The number of inappropriate prophylactic antimicrobial timing cases decreased from 55 cases/year at baseline to approximately 35 cases/year in the second phase and approximately 34 cases/year in the third phase, while the appropriate timing rate increased from 99.23 to 99.56% (second phase) and 99.66% (third phase);
- The number of inappropriate prophylactic antimicrobial course cases decreased from 21 cases/year at baseline to approximately 13 cases/year in the second phase and approximately 9 cases/year in the third phase, with the appropriate course rate rising from 99.71 to 99.84% (second phase) and 99.91% (third phase);
- The number of inappropriate prophylactic antimicrobial agent selection cases decreased from 13 cases/year at baseline to approximately 8 cases/year in the second phase and approximately 3 cases/year in the third phase, and the appropriate agent selection rate increased from 99.71 to 99.84% (second phase) and 99.97% (third phase) (Table 6).

**Table 6:** Summary of rational use of antimicrobial prophylaxis in Class I incision surgeries from 2017 to 2024.

Time period	Class I incision surgery cases	Prophylactic antibiotics	Number of cases with unreasonable medication timing	Reasonable rate of medication timing	Number of cases with unreasonable medication courses	Medication treatment course rationality rate (%)	Number of cases of irrational drug use	Rate of rationality in drug variety (%)
Baseline data	21,259	7,168	55	99.23	21	99.71	13	99.82
Phase I	48,608	15,038	114	99.24	23	99.85	23	99.85
Phase II	83,804	24,314	106	99.56	40	99.84	19	99.92
Phase III	76,259	19,757	68	99.66	17	99.91	5	99.97

### Class I incision site infection conditions

As shown in Table 7, after the implementation of the administrative Multi-disciplinary Team (MDT) intervention, the incidence of surgical site infections (SSI) in Class I incision surgeries decreased from 0.11% in 2017 to 0.09% in the second phase (χ^2^ = 0.727, *p* = 0.394 > 0.05) and 0.10% in the third phase (χ^2^ = 0.034, *p* = 0.855 > 0.05). Overall, there was a downward trend, but the differences were not statistically significant according to statistical tests.

**Table 7:** Statistics of incidence rate of surgical site infections for Class I incisions from 2017 to 2024.

Time period	Class I incision surgery cases	Number of infection cases	Incidence rate	Χ^2^	*p*
Baseline data	21,259	23	0.11		
Phase I	48,608	64	0.13	0.655	0.42
Phase II	83,804	74	0.09	0.727	0.39
Phase III	76,259	78	0.13	0.034	0.855

Chi-square tests were performed to compare the first, second, and third phases with the baseline data.

## Discussion

### The knowledge and theoretical understanding of perioperative antimicrobial application among surgeons show an upward trend

Studies have confirmed that through the implementation of hospital-wide training on antimicrobial stewardship knowledge, together with specialized exchanges carried out by medical, pharmaceutical, and infection control departments via joint participation in departmental morning handovers, ward rounds, and academic activities, surgeons’ mastery of knowledge regarding perioperative prophylactic antimicrobial use in Class I incisions showed a significant upward trend. The average score rate increased from 70.83% in 2017 to 96.33% in the second stage and 95.41% in the third stage, which verified the effectiveness of graded and multi-form training (7–10). Affected by the COVID-19 pandemic since 2020, all hospital training has been converted to online training and assessment, and the impact of this mode on surgeons’ knowledge mastery needs further verification. In addition, knowledge scores can only serve as an auxiliary reference indicator for the improvement of medical staff’s cognition. Future research should combine knowledge mastery with clinical practice (rationality of antimicrobial use) for comprehensive evaluation and analysis.

### Significant reduction in prophylactic antimicrobial use rate for perioperative period of Class I incision surgeries

In the first phase, with the improvement of surgical doctors’ mastery of perioperative-related knowledge, relevant functional departments actively carried out monitoring, supervision, and feedback (11–13), leading to a substantial improvement in the prophylactic use rate of antimicrobial agents for perioperative period of Class I incisions. In the second phase, with the involvement of nursing teams, operating room teams, and anesthesiology teams, clinical doctors received better reminders regarding medication timing, course, and other aspects (14, 15). As a result, the prophylactic use rate of antimicrobial agents for perioperative period of Class I incisions decreased from 33.72% in 2017 to 29.01% in the second phase. Statistical analysis showed that the difference was statistically significant (χ^2^ = 178.84, *p* = 0.000 < 0.05), indicating a marked improvement. Continuous tracking of the improvement in perioperative prophylactic medication use and good multi-department collaboration resulted in a prophylactic antimicrobial use rate of 25.91% in the third phase, which was also statistically significant (χ^2^ = 507.31, *p* = 0.000 < 0.05).

Statistical analysis of the improvement in prophylactic antimicrobial use cases and rates among the top ten departments with the highest comprehensive rankings revealed that the prophylactic use rates in Cardiac Surgery, Neurosurgery, Trauma Orthopedics, Ophthalmology, and Urology decreased significantly, with all differences being statistically significant. This indicates that under the premise of unchanged overall surgical composition, the intervention of the administrative Multi-disciplinary Team (MDT) had a marked effect, and clinical doctors could more accurately grasp the indications for antimicrobial use, reducing the prophylactic use rate of preoperative antimicrobial agents (16). In recent years, the surgical volume of Gynecology has decreased, but the awareness of perioperative prophylactic antimicrobial use has generally improved, which is basically consistent with relevant studies (17). The prophylactic use rates of antimicrobial agents for perioperative period in Bone and Joint Surgery, Spine Surgery, and Breast and Thyroid Surgery showed a trend of first increasing and then decreasing. The main reasons are the rapid development of the hospital since 2018, with increases in surgical volume and difficulty, as well as an increase in patients over 65 and those with underlying diseases such as diabetes.

The launch of the new campus (adding 1,500 beds) in mid-2020 led to no significant change in the second phase compared to the baseline. However, with the collaborative participation of multiple departments in the management of perioperative prophylactic antimicrobial use, the standardization of perioperative prophylactic antimicrobial use showed a clear upward trend, and the improvement effect was significant in the third phase according to departmental monitoring data (9, 18). Compared with relevant domestic studies, the prophylactic use rate of antimicrobial agents for perioperative period in the top ten departments of our hospital remains at a relatively low level (19). Another key issue to focus on is that the surgical volumes of Ophthalmology and Breast and Thyroid Surgery, which have lower preoperative prophylactic antimicrobial use rates, are showing a clear upward trend (20, 21), and their large proportion is also a reason for the low overall antimicrobial use rate in the hospital.

### The rationality of prophylactic antimicrobial use in the perioperative period of Class I incision surgeries showed a year-by-year upward trend

(1) Issues with inappropriate timing: Some doctors had insufficient understanding of perioperative medication, and under the condition of increased surgical volume, they compressed the turnover time between operations to ensure the smooth progress of surgeries. This led to situations where preoperative medication had not been fully infused before the operation started, or emergency situations caused the infusion to not be completed within 0.5–1 h before surgery. (2) Issues with inappropriate duration: Inadequate handover among clinical doctors resulted in delayed discontinuation of antimicrobial orders, leading to prolonged treatment courses. Additionally, failure to adjust the purpose of medication in a timely manner when patients developed infection signs postoperatively also contributed to extended courses. (3) Main issues with inappropriate drug selection: Some doctors did not strictly adhere to the requirements of the Operational Procedures for Perioperative Prophylactic Use of Antimicrobial Agents or had inadequate assessment of patients’ conditions, resulting in the off-class use of antimicrobial agents. A critical research revelation demonstrates that operating room nurses, through systematic verification protocols, can effectively detect anomalies in medication administration timing and pharmacological agent selection, subsequently implementing corrective interventions to mitigate potential adverse events.

### The incidence of surgical site infections (SSI) in Class I incision surgeries showed a year-by-year decreasing trend

For perioperative prophylactic antimicrobial use in Class I incisions, decisions on whether to administer prophylactic antimicrobial agents should be made comprehensively based on factors such as the degree of surgical trauma, possible contaminating bacterial species, surgical duration, opportunities and severity of infection, and evidence-based medical evidence for the preventive effect of antimicrobial agents. This study demonstrated that after the intervention of the administrative Multi-disciplinary Team (MDT), the prophylactic use rate of antimicrobial agents in the perioperative period of Class I incisions showed a significant decreasing trend, while maintaining the SSI incidence at a low level (22–24).

## Conclusion

This study demonstrated that the administrative Multi-disciplinary Team (MDT) model effectively integrated regulatory and clinical departments (including medical, nursing, and infection control teams) to optimize perioperative antimicrobial prophylaxis for Class I incision surgeries. Key interventions included:

1 Structured training programs: Comprehensive training (hospital-wide, targeted, and thematic) enhanced surgeons’ knowledge of antimicrobial use guidelines.
2 Standardized protocols: A flowchart-based perioperative workflow clarified roles across departments:
• Preoperative: Surgeons assessed antimicrobial necessity and selection; operating room nurses administered preoperative infusions.
• Intraoperative: Surgeons, anesthesiologists, and nurses jointly evaluated intraoperative supplementation.
• Postoperative: Surgeons determined prophylactic duration; nurses monitored compliance.
3 Continuous monitoring: Monthly audits, data transparency, and penalties for non-compliant cases reduced misuse.

These measures significantly reduced the prophylactic antimicrobial use rate (from 33.72 to 29.01%) while maintaining a low Surgical Site Infection (SSI) incidence rate (0.09–0.10%), validating the effectiveness of administration-driven multidisciplinary teams (MDT) in rationally reducing unnecessary antimicrobial use.

### Prospects

Four areas require sustained improvement:

1. Enhanced information systems: Implementing AI-driven antimicrobial stewardship platforms (e.g., automated alerts for off-guideline prescriptions) could further refine decision-making, as shown in studies optimizing electronic review systems (25, 26).
2. Behavioral change interventions: Persistent gaps in clinicians’ awareness (e.g., overestimation of prophylactic necessity) warrant tailored workshops and performance-linked incentives.
3. Protocol refinement: Developing department-specific targets (e.g., surgery Class-adjusted thresholds) and incorporating compliance metrics into department heads’ annual evaluations could strengthen accountability.
4. Interdisciplinary collaboration: Expanding roles for operating room nurses and anesthesiologists in antimicrobial stewardship (e.g., real-time dose adjustments) aligns with evidence from multi-center trials emphasizing team-based care (27–29).

### Limitations

In this study, comparisons of age distribution, diabetes status, and whether surgery duration exceeded 3 h across different stages showed no statistically significant differences. However, several unresolved potential time-related confounding factors (such as policy changes and variations in surgeon skill levels) remain (30) and this is an “observational before-after study without concurrent controls,” and thus cannot establish a causal relationship between the MDT intervention and outcomes, it can only reflect an association and temporal correlation.

## Data Availability

The raw data supporting the conclusions of this article will be made available by the authors, without undue reservation.
